# Orthodontic aligners: between passion and science

**DOI:** 10.1590/2177-6709.28.6.e23spe6

**Published:** 2024-01-05

**Authors:** Suelly Maria MENDES RIBEIRO, Mônica Lídia Santos de Castro ARAGÓN, Daybelis del Socorro González ESPINOSA, Wendel Minoro Muniz SHIBASAKI, David NORMANDO

**Affiliations:** 1 Universidade Federal do Pará (UFPA), Faculdade de Odontologia, Doutorado em Odontologia (Belém/PA, Brazil).; 2 Universidade Federal do Pará (UFPA), Faculdade de Odontologia, Mestrado em Ortodontia (Belém/PA, Brazil).; 3 Universidade Estadual Paulista (UNESP), Doutorado em Ortodontia (Araraquara/SP, Brazil).; 4 Universidade Federal do Pará (UFPA), Departamento de Ortodontia (Belém/PA, Brazil).

**Keywords:** Orthodontic aligners, Removable orthodontic appliance, Tooth movement, Biomechanics, Malocclusion, Quality of life, Root resorption, Sustainability, Biocompatibility, Aesthetics

## Abstract

**Introduction:**

The benefits and safety of using orthodontic aligners have been reported more by clinical experience and expert opinion than by scientific evidence. Another important aspect is that aligners are constantly evolving. It is important to obtain evidence that allows for new updates in manufacturing technology, in the development of new movement planning protocols, in the incorporation and design of attachments, and in the aid of skeletal anchorage.

**Methods:**

Evidence retrieved from six electronic databases (CINAHL, MEDLINE, EMBASE, Psych Info, the Cochrane Library and the Joanna Briggs Library) is presented by means of questions and answers.

**Conclusions:**

There is evidence that the aligners presented different levels of difficulty in performing each type of movement, with rotational and vertical movements being the most difficult to perform. Regarding perception of pain due to tooth movement, it seems to have less impact at the beginning of treatment; but dealing with more phonoarticulatory changes seems to require more treatment time in more complex cases. Aligners do not prevent the occurrence of root resorption, although the incidence and severity of resorption may be reduced, making oral hygiene easier and accepting the risk of white spots, caries and periodontal disease. Given the conflicting evidence, the release of bisphenol-A from the aligner cannot be denied. Solutions must be found to reduce the environmental impact of aligners disposal. There is an urgent need for well-designed randomized controlled trials.

## RESUMO

**Introdução:**

As vantagens e desvantagens do uso de alinhadores ortodônticos têm sido reportadas com base mais na experiência clínica e opinião de *experts* do que em evidências científicas. Outro aspecto importante é que os alinhadores estão em processo de evolução constante. Assim, torna-se importante obter evidências recentes, que abranjam as novas atualizações na tecnologia de confecção, no desenvolvimento de novos protocolos de planejamento para as movimentações, a incorporação e desenho dos *attachments* e o auxílio da ancoragem esquelética.

**Métodos:**

As evidências encontradas em seis bases de dados eletrônicas (CINAHL, MEDLINE, EMBASE, Psych Info, Biblioteca Cochrane e Biblioteca Joanna Briggs) serão apresentadas por meio de perguntas e respostas.

**Conclusões:**

Há evidências de que os alinhadores apresentam diferentes níveis de dificuldade para realizar cada tipo de movimento, sendo os movimentos rotacionais e verticais os mais difíceis de serem executados. Quanto à percepção da dor causada pela movimentação dentária, parece haver menos impacto no início do tratamento, mas os alinhadores produzem mais alterações fonoarticulatórias e parecem exigir mais tempo de tratamento em casos mais complexos. Não há evidência de diferença na estabilidade pós-tratamento, e os alinhadores não impedem a ocorrência de reabsorção radicular, apesar da incidência e a gravidade da reabsorção poderem ser menores, facilitam a higiene bucal, reduzindo o risco de manchas brancas, cáries e doenças periodontais. Dada a evidência conflitante, a liberação de bisfenol-A pelo alinhador não pode ser negada. É preciso encontrar soluções que reduzam o impacto ambiental do descarte dos alinhadores. Há uma necessidade urgente de ensaios clínicos randomizados bem desenhados.

## INTRODUCTION

Orthodontic aligners have gained a lot of visibility in recent years, but it is important to know their real effects in detail, rather than just believing industry’s passionate marketing pitch.

It is well known that systematic reviews are the best scientific evidence on a given clinical question. In 2005, when the first systematic review on aligners effectiveness was published,^1^ it was not possible to compile consistent scientific evidence on the indications, limitations and effectiveness of orthodontic treatment with aligners. There was a lack of reliable clinical trials evaluating the planning and effects of treatment with these devices. Since then, about 30 systematic reviews have been published, and they are necessary due to the many changes in manufacturing technology and orthodontic treatment systems using aligners, combined with the accumulation of clinical and scientific knowledge. It is therefore necessary to ask: where do we stand today?

**Figure 1:**
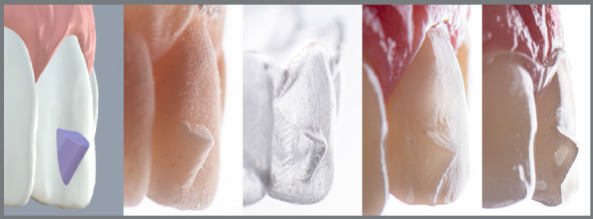
Planning and incorporation of attachments to increase the effectiveness of movements produced by orthodontic aligners.

**Figure 2:**
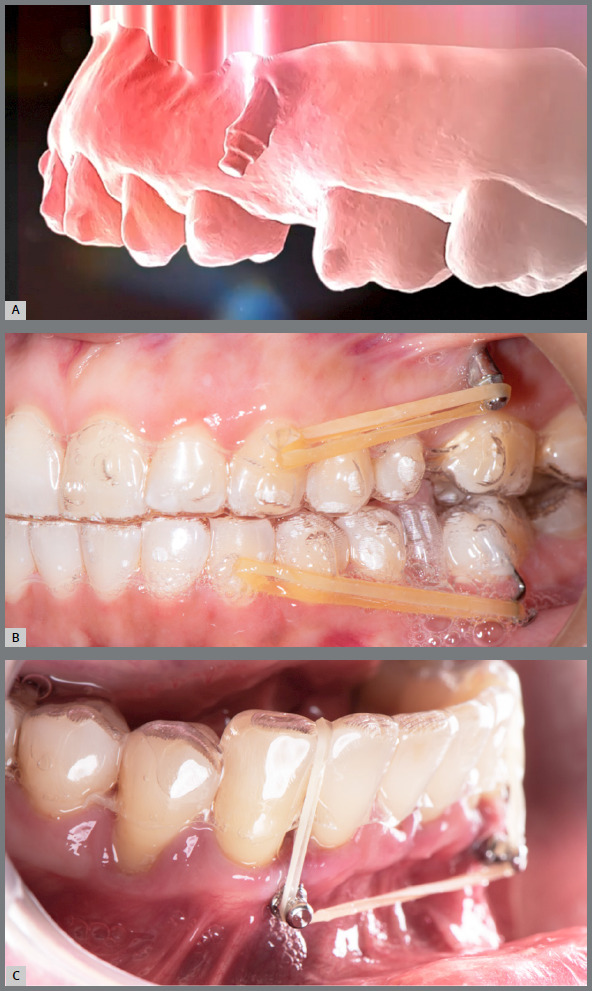
A) Skeletal mini-implant in the upper arch. B) Aligners installed with auxiliary elastics. C) Skeletal mini-implant in the lower arch with auxiliary elastics.

Only in 2022, more than 150 scientific articles were published on orthodontic aligners. Despite this significant number, indications, advantages and disadvantages have been reported based on clinical experience and expert opinion rather than scientific evidence.^2^ ^-^ ^4^ Another important aspect is that aligners are constantly evolving: an aligner studied in 2005 may not have the same characteristics of an aligner used in 2023. Therefore, it is important to obtain recent data, including new updates in aligner manufacturing technology, the development of new movement planning protocols,^5^ the incorporation and design of attachments (Fig 1) and skeletal anchoring devices^6^ ^,^ ^7^ (Fig. 2). 

Constantly, some companies have propagated numerous advantages of orthodontic aligners in relation to fixed appliances. It is claimed that aligner treatment would be faster, cause less pain/discomfort and align teeth more predictably.^8^ Some of these assumptions are even passionately publicized. But what would be science’s response to these claims? Thus, the objective of this article is to compile, in a broad but objective way, the most current scientific evidence, answering several clinical questions about orthodontic treatment with aligners, in contrast to what is propagated by industry marketing. It is expected, however, that the results of this review do not allow conclusions about the latest updates in this treatment system, due to the constant technological evolution. Thus, when reading this article, the clinician should be aware that material tested in a scientific study may already have been replaced or modified. However, the evolution of a material does not qualify it as more efficient than the previous one.^9^ It is worth remembering that most of the current advantages propagated by the industry have already been disclosed in the past and have not been confirmed by science.

## 1. CAN ALIGNERS PERFORM ALL TYPES OF ORTHODONTIC MOVEMENTS?

The primary function of an orthodontic appliance is to move teeth in three directions of space. The effectiveness of aligners in performing some types of orthodontic movements has been investigated by comparing the movement predicted in the virtual planning with the one actually obtained after treatment or at the end of a set of aligners.^10^ Due to the diversity of movements performed in orthodontics and the heterogeneity of results, an individual analysis of each type of movement is necessary.

### A. TRANSVERSE MOVEMENTS OF THE DENTAL ARCH

There is a clinical consensus that orthodontic aligners would be an effective tool to promote transverse expansion of the dental arches. According to a systematic review published in 2018, including 20 studies, aligners seem to be effective in increasing inter-canine, inter-premolar, and inter-molar width in the presence of crowding.^3^ In the past two years, six primary studies have been published looking at the predictability of transverse movements with aligners. Therefore, this subject deserves an update. In summary, the expansion movement demonstrates good accuracy, comparing the movement obtained at the end of the treatment with that predicted in ClinCheck.^11^ ^,^ ^12^ The dental element with greater accuracy does not seem to be unanimous, due to the heterogeneity of the methodology and objectives of the studies, varying between premolars^11^ ^,^ ^13^ and first molars.^12^

A more recent study retrospectively evaluated 57 adult patients and collaborators who received orthodontic treatment with the Invisalign^®^ system and whose planning included a transverse expansion greater than or equal to 3 mm.^13^ The predictability of expansion in the area of maxillary canines and premolars was between 70-80% and, surprisingly, it was a little higher for the lower arch (80-90%) and smaller in the area of ​​the first molars, reaching a little more than 40% for the second molars. Interestingly, inaccuracy can be caused by overexpansion, not just the undercorrection often seen in other types of movements with aligners. This can be corroborated by the fact that we found articles reporting that, in relation to the amount of expansion, the maxillary first molars^14^ and the mandibular second molars are the teeth that present the greatest expansion at the end of the treatment.^11^ It became clear that expansion with aligners can be achieved, even in adult patients, although it is critical in the distal region of the arch, in molars. Technological development should therefore focus on improving the transverse effects at the ends of the arches, perhaps increasing the rigidity of the plastic in this region. When considering treatment in the mixed dentition phase, with the Invisalign First^®^ system, aligners can be considered effective in growing patients who require dimensional increase in the upper arch. The most significant transverse increase occurred at the level of the maxillary deciduous first molars, while the maxillary first permanent molars showed a greater expansion in the mesial intermolar width due to the rotation that occurs around their palatal root.^15^ ^-^ ^17^ However, there is little evidence about the benefits of expansion achieved with aligners when compared to rapid maxillary expansion. 

A study published in 2022^12^ found statistically significant morphological changes in the shape of the upper arch, with an increase in the transverse dimension in the anterior region at the level of the canine and the first deciduous molar widths in individuals treated with Invisalign First^®^, when compared to individuals who used a rapid maxillary expander. However, the expander used in the study was a butterfly-type modification of the Hyrax, fixed only on molars, without arms or extensions for premolars or canines.

### B. DENTAL ROTATION MOVEMENT

Unlike the expansion movement, current evidence indicates that orthodontic aligners are not effective in performing rotational movements , especially of rounded teeth. This was one of the conclusions of a systematic review published in 2015.^6^ Later, these data were complemented by another systematic review, published in 2018,^2^ in which it was concluded that the aligners are capable of rotating teeth, except canines and premolars.

It is unanimous that rotations are movements that are difficult to achieve with aligners. Therefore, it is suggested that interproximal wear be carried out, and that rotations greater than 1.5º per aligner are not performed.^3^ When rotations greater than 1.5º are necessary, additional resources should be used, such as attachments or even mini-implants and elastics supported by buttons and hooks.^7^

Some studies have emphasized that the potential for teeth movement must be analyzed separately, as they exhibit specific radicular characteristics and morphological features of the crown, as well as the histology of the alveolar bone support. These issues must be considered when analyzing the individual response of teeth to orthodontic forces.^17^ ^,^ ^18^

More recently, in 2023, a prospective clinical study investigated the predictability of tooth movements performed with aligners.^19^ Differences were found between predicted and achieved results in all six movement categories analyzed: angulation, inclination, rotation, and mesiodistal, vertical, and buccolingual movements. This study corroborated that the rotation of maxillary lateral incisors, canines and premolars is difficult to obtain in view of what was foreseen in the planning, and concluded that the only rotation movement with excellent predictability is that of the first permanent molars. Further refinements are needed in almost all cases to overcome the limited predictability of aligner treatment for most movements.

### C. DENTAL VERTICAL MOVEMENTS

Along with rotations, vertical movements are considered the most challenging movements to be achieved with aligners.^3^ Extrusion movement is harder to control, with only 30% predictability. It is known that aligners cannot effectively control anterior extrusion.^6^ Regarding posterior vertical control, there seems to be no conclusion.^6^ Until 2020, the predictability of extrusion of anterior teeth had a very low level of evidence, although it increased in comparison with the conclusions of previously published systematic reviews.^4^

More recently, in 2022, a clinical study^20^ evaluated the accuracy of deep overbite correction by comparing the predicted result of ClinCheck^®^ with the result achieved after treatment for groups that used different technologies of the aligner system: EX30 without bite ramps *versus* SmartTrack with precision bite ramps, released in 2013. For both groups, the deeper the patient’s initial overbite, and the greater the amount of correction predicted by ClinCheck^®^, the greater the difference in relation to the result obtained. While ClinCheck^®^ predicted an overbite reduction of 95.3%, the correction actually achieved was only 39.2%. Thus, the presence of a deeper initial overbite reduces the treatment effectiveness.

A recent study, published in 2023, confirmed that at the end of the first sequence of aligners, only 33% of the overbite correction was achieved, or 1.15 mm of reduction, on average.^19^ The authors suggested that overcorrections and further refinements are needed in most deep bite patients. Another more feasible possibility would be the use of skeletal anchorage and sequential movements. Apparently, the vertical movements are more effective for the lower incisors, and of less effective control in premolars and molars in both arches, as reports the recent literature on the subject.^19^

With regard to open bite, a retrospective study analyzed the effectiveness of aligners in correcting surgical and non-surgical cases, and reported success in 94% of patients. The mean total overbite change was 3.3 ± 1.4 mm, demonstrating that aligners can be effective in controlling the vertical dimension and correcting mild to moderate anterior open bite in non-extraction adults. Changes were observed in terms of retroclination of the upper incisors and intrusion of the upper molars, reaching an overbite of 1.1 ± 0.8 mm at the end of the treatment. However, the factors that most contributed to the correction were the extrusion of the upper and lower incisors and the reduction of the mandibular plane.^21^ The authors did not discuss the effect of incisor retroclination in the treatment of overbite. These findings confirm similar previously published results.^22^

### D. DENTAL ANTEROPOSTERIOR MOVEMENTS

In the meantime, aligners can produce clinically acceptable results that may be comparable to fixed appliance therapy, as reported in a systematic review published in 2020.^4^ These results are mainly related to buccolingual inclination movements of the mandibular incisors in mild to moderate malocclusions.^23^ However, despite recent advances, most tooth movements may not be predictable enough to be accomplished with just one set of clear aligners, as described earlier.^4^

The aligners seem capable of controlling the movement of the upper molar body when a distalization of 1.5 mm is prescribed.^6^ However, lower molars are teeth that are difficult to control.^19^ According to the literature, it is not necessary to incorporate attachments in mechanics to perform molar distalization, whose movement accuracy would be approximately 88%^3^. Complementing this conclusion, a prospective study published in 2023 reported that lower molars are the teeth with the lowest response, and almost always inexpressive.^19^ It is noteworthy that in this study, approximately 60% of the second molars did not receive attachments. The authors consider that when moving from the center to the distal ends of the aligners, their elasticity increases, so that it can be speculated that the use of attachments in distal teeth can increase the rigidity of the system, benefiting the control of angulation and inclination movements.^23^

**Figure 3:**
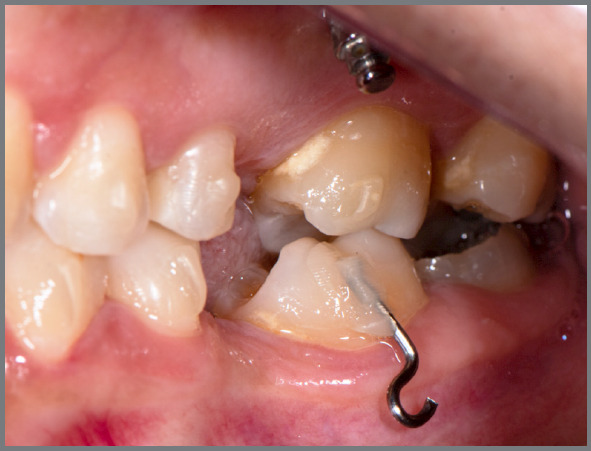
Auxiliary resources to increase the effectiveness of movements with orthodontic aligners, such as: attachments, skeletal anchorage, power arms.

**Figure 4:**
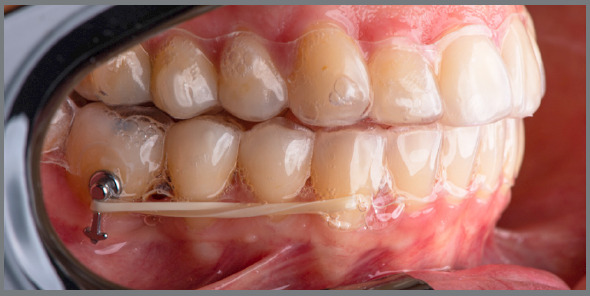
Elastic bands associated with power arms as auxiliary resources to increase the effectiveness of movements with orthodontic aligners.

Despite existing limitations, evidence shows that aligners are capable of aligning dental arches.^2^ ^,^ ^6^ In fact, some movements, such as root movements, seem to be better controlled with fixed appliances.^3^ Teeth angulations and occlusal contacts seem to be among the challenges of aligner treatment when it comes to the accuracy of planned movements.^2^ ^,^ ^3^ ^,^ ^15^ To overcome this limited predictability of current therapy with clear aligners, further refinements are needed in most cases.^4^ The use of auxiliary resources such as attachments , skeletal anchorage, power arms (Fig 3) and elastics (Fig 4) is described to improve the effectiveness of this treatment modality.^3^

## 2. CAN ALIGNERS TREAT COMPLEX CASES?

According to the pioneering company in the manufacture of aligners, currently this device could effectively perform large dental movements and treat several changes in the dentition, such as crowding, overbite, prognathism, crossbite, diastema, open bite, whether in the deciduous and/or permanent dentition.^8^ However, few studies evaluated the current scientific evidence regarding the effectiveness of treating more complex cases with aligners.

In this environment of doubts, a systematic review published in 2018, including 22 articles, concluded that ideally, aligners should be indicated for cases of mild to moderate malocclusion without extractions^2^. This indication was ratified in an overview of systematic reviews published in 2022.^10^

Only one randomized controlled clinical trial^5^ compared the efficiency of aligners with fixed appliances in treating complex cases. This study considered as complex cases the presence of Class I malocclusion with 6 mm of crowding, and concluded that the in-office aligners can obtain satisfactory results when treating complex cases using the tooth movement protocol entitled SAMMER (Stepwise Activated Movements by Multiple Enhanced Re-anchorage). This protocol divides tooth movements into three stages and defines 0.25mm as the maximum limit of tooth movement per aligner for transitional movements, and 2° for rotations. Attachments can be added where needed. This study considered the need for extraction of premolars to resolve the complexity of malocclusion and a greater number of aligners.

## 3. DO AUXILIARY DEVICES INCREASE THE EFFICIENCY OF ALIGNERS?

Considering that the aligners manage to move the teeth through the pressure transmitted by the plastic to the tooth, some studies have evaluated the inclusion of auxiliary resources such as attachments, interproximal wear, elastics and altered geometry of the aligners^7^ ^,^ ^24^ (Fig 4). The authors conclude that these devices are necessary to obtain the planned results.

A recent systematic review, published in 2022, showed that success in tooth movement using dental aligners can be especially influenced by the use of attachments,^7^ which are composite resin buttons bonded to the tooth surface with the aim of improving retention and facilitate the most complex movements.^25^ When selecting this type of auxiliary device, several factors must be taken into account, such as correct location, selection of shape, size and number of attachments, as they can directly interfere with the efficiency of dental movement. The restrictions of this review are related to the limitations of the included studies, causing a great heterogeneity in the results.^7^ The most used attachment formats are horizontal, vertical and bevelled.^25^ ^,^ ^26^ ^,^ ^27^ The extrusive movements are difficult to execute, due to the lack of elastic deformation of the alignment in the vertical direction. Therefore, it is necessary to use horizontal attachments and sometimes complement them with the use of accessories bonded to the palate surface of the teeth, to guide the extrusion of the incisors with the aid of elastics.^7^ ^,^ ^24^ ^,^ ^25^ In addition, this type of accessory is also used on premolars to increase control retention when using Class II or III elastics, and to correct Spee’s curve and deep bite.

When using an aligner system, it must be taken into account that the attachments do not necessarily provide anchorage to teeth in all cases. It is crucial for an efficient treatment to know the type of material, the thickness, and the proper biomechanics, before combining it with different attachments.^26^ ^,^ ^27^

Introduced more recently, the “power ridges” are attachments that generate a negative bubble in the model, which, after plasticizing the aligner, works as a pressure area. They were initially designed just to improve root control in incisor tipping movements, by putting extra pressure on the root of the tooth. Recently they have been extended to improve torque correction (>3°) and vertical control of the incisor axis, and also in simultaneous retraction and extrusion movements.^28^

In the corrective treatment of moderate malocclusions, it is common to complement the treatment with intermaxillary elastics, especially in mild Class II cases. This approach can amplify the overbite correction and prevent undesirable inclination of the lower incisors, in addition to providing better control of the upper incisors during the retraction process.^29^

Due to the limitations of using elastics, in more complex cases, the use of temporary skeletal anchorage is necessary, especially due to the horizontal and vertical component for sagittal correction, which can displace the aligner. The application of anchorage devices is more common in cases of open bite, Class III and Class II. Orthodontic mini-implants can also be used in hybrid treatments, adding components of the fixed appliance treatment, with the aim of amplifying skeletal maxillary expansion in Class III malocclusion.^30^

## 4. IS TREATMENT WITH ALIGNERS MORE EFFICIENT THAN WITH FIXED APPLIANCE?

A systematic review published in 2018^2^ identified a limited effectiveness for orthodontic treatment with aligners, mainly in movements such as the expansion of the dental arch through bodily movements of the teeth, in closing the extraction space, in correcting occlusal contacts and in cases of large anteroposterior and vertical discrepancies. The Invisalign^®^ system can treat non-extraction cases and mild malocclusion more quickly, but requires more treatment time than fixed appliances for more complex cases.

Publications up to 2019 were analyzed in a systematic review^4^, and the studies presented a ‘low to moderate level’ of certainty regarding the efficiency of tooth movements produced by aligners in the face of different types of malocclusion. However, not all possible clinical scenarios have been considered. One year later, a systematic review associated orthodontic treatment with aligners with worse treatment outcomes compared to fixed appliances.^31^

Subsequent to the publication of these systematic reviews, in 2021, a study with conflict of interest analyzed the occlusal improvement of patients treated with aligners, and demonstrated that these devices were able to significantly reduce malocclusion in adult patients without extractions. The observed improvement rate was 77.44%, considering that values above 70% suggest a good result.^32^

When compared to the positive changes in the PAR index, between complex cases treated with in-office aligners and fixed appliances, both systems showed good completion.^5^ This randomized clinical trial found no significant difference in treatment duration between the studied groups. Apparently, this is a very controversial subject. The results differ from data obtained in a previous study,^32^ in which treatment with Invisalign^®^ aligners was 44% longer than treatment with fixed appliances in cases of extraction of four premolars, associated with worse results compared to fixed appliances.

Regarding mixed dentition, a randomized clinical study published in 2022 concluded that patients treated with aligners and 4x2 fixed mechanics showed similar efficacy and efficiency for the correction of crowding of the upper incisors. Similar results of post-treatment arch alignment were observed and the treatment time was approximately 8 months in both groups.^34^

Duration of treatment is another issue that deserves attention. A clinical study comparing aligners with fixed appliances in cases of premolar extraction showed that both approaches produced satisfactory results, including space closure and effective anchorage control, but treatment time was longer with aligners.^32^ This difference in treatment time was related to the fact that Invisalign patients require up to seven months of finishing. Other studies have shown that there is no difference in treatment time between aligners and fixed appliances.^5^ ^,^ ^23^ ^,^ ^31^ ^,^ ^33^ However, it does not seem to be determined by the type of appliance alone, as factors related to the patient or treatment must be considered^31^ in addition to the biomechanics used.^2^ A key issue in the effectiveness of aligners remains patient compliance and cooperation with treatment.

## 5. CAN ORTHODONTIC TREATMENT WITH ALIGNERS IMPACT ON THE ORAL HEALTH-RELATED QUALITY OF LIFE?

One of the goals of orthodontic treatment is to improve people’s quality of life through less invasive and more comfortable methods. Fixed orthodontic treatment has been an excellent tool in the treatment of various types of malocclusion, but it can have a negative impact on quality of life 24 hours after appliance placement and up to three months after treatment initiation.^35^ ^,^ ^36^

A systematic review published in 2020^37^ evaluated the impact of aligners on quality of life, and concluded that during and after treatment, aligners are able to provide a better quality of life by causing less chewing and eating discomfort, compared to fixed appliances. However, these results may be explained by the fact that aligners are removable devices and the patient can remove them while eating, and that they are more comfortable to wear because they have smooth surfaces and edges.^37^

When comparing the psychosocial, environmental and physical health factors that may influence the use of orthodontic aligners, lingual orthodontics and conventional fixed orthodontics, it was observed that patients undergoing treatment with aligners reported the best quality of life scores, followed by the lingual orthodontics group and, lastly, the fixed orthodontics group.^38^ It should be borne in mind that sociocultural perceptions may vary in different parts of the world. Dental aligners may provide a better perception of orthodontic treatment for the patient and his or her surroundings, as it is the most aesthetic treatment on the market that favors the way the patient can relate socially, and may even gain greater importance in the era of social media. However, these results may be influenced by confounding factors, such as the severity of the malocclusion.^37^

Another confounding factor is the difference in each individual’s perception of pain, since physical pain is considered one of the most influential factors on the quality of life of patients undergoing orthodontic treatment. A systematic review found that patients treated with aligners reported lower levels of pain compared to those treated with fixed appliances during the first days of treatment, but there was no difference throughout treatment.^39^ These results have been confirmed by clinical studies published subsequently,^40^ including a prospective clinical study.^41^

It is common that during orthodontic treatment patients experience some discomfort during the adaptation process, because the devices cause pain or even interfere with tongue movements, limiting the space for tongue accommodation and therefore the patient’s speech.^42^ ^,^ ^43^ ^,^ ^44^

Because they are plates that cover the tooth surface, aligners may cause discomfort to the patient in verbal articulation.^38^ Several studies have reported that esthetic aligners can influence the clarity and articulation of speech, in a more pronounced way than fixed appliances.^37^ ^,^ ^41^ These interferences are mainly errors in the articulation of sibilant sounds and alveolar fricative consonants (s and z), and interference in primary phonemes (including /s/, /z/, /zh/, /sh/, /th/, /ch/). It has also been reported that these speech difficulties were temporary and most patients were able to adapt around 7 to 14 days from the start of treatment, while a few patients took up to 60 days for normalization.^41^ ^,^ ^45^ These interferences should be informed to the patient before the start of treatment with orthodontic aligners.^46^ ^,^ ^47^

## 6. WHAT IS THE REAL AESTHETIC ACCEPTABILITY OF ALIGNERS?

To date, little research has been conducted to assess patients’ perceptions of the appearance of aesthetic appliance systems such as aligners, with or without attachments. In one study, photographs of patients smiling with aligners, smiles with aligners and attachments on posterior teeth only, aligners with attachments on anterior and posterior teeth, and smiles of patients wearing ceramic brackets were evaluated using eye tracking.^48^ The results indicated that although adult patients desire aesthetic appliances, what is considered aesthetic varies by demographic, and many prefer the appearance of minimal accessories and ceramic brackets to aligners with multiple attachments. Therefore, in patients with complex movements that require many attachments, it may be more appropriate to use ceramic brackets.

One study evaluated the satisfaction of patients who completed treatment with aligners in relation to their appearance, and found moderate to high levels of satisfaction. This suggests that the aesthetic acceptability of aligners is a consideration in the patient’s choice of device.^49^ In a descriptive cross-sectional study conducted using questionnaires assessing the perception and satisfaction of patients treated with orthodontic aligners,^50^ it was observed that the main reason for choosing this treatment modality was mainly its “invisibility” ( 89.7%), and 58.8% of patients would be satisfied with the aesthetics of the aligners.

In the meantime, orthodontists should demonstrate and discuss with patients the various options for orthodontic appliances so that the decision is centered on the patient.

## 7. DOES TREATMENT WITH ORTHODONTIC ALIGNERS RESULT IN GREATER STABILITY AFTER TREATMENT?

There is little evidence on the stability of aligner treatment compared to conventional fixed appliance therapy.^1^ A systematic review and meta-analysis evaluated the efficiency, efficacy, and stability of clear aligner treatment compared with conventional bracket treatment in four controlled clinical trials involving a total of 252 participants^51^. The authors noted that reduced treatment time and chair time in mild to moderate cases appeared to be the only evidence in favour of aligners over conventional systems. No difference in stability and occlusal characteristics after treatment was found between the two systems.^51^

## 8. DOES TREATMENT WITH ORTHODONTIC ALIGNERS CAUSE LESS ROOT RESORPTION THAN FIXED APPLIANCES?

Preserving tooth structure during orthodontic movement is one of the main concerns of orthodontists. A systematic review published in 2017 found that cases of mild to moderate malocclusion without the need for extractions treated with aligners may be associated with a lower incidence of root resorption than treatment with fixed appliances. Research on extraction cases is needed to better assess the incidence and severity of root resorption following the use of these removable appliances.^52^

A systematic review with meta-analysis published in 2019 suggests that external root resorption was significantly lower in treatments with clear aligners than with fixed appliances, and that aligners may not prevent root resorption, but the incidence and severity of resorption may be less compared to the results reported with treatment with fixed appliances.^53^

In 2021, a study justified this lack of significant change by stating that aligners absorb less energy because they permanently deform under moderate to heavy loads and have significantly less elasticity compared to metal archwires with memory. Clinically, crowding that can be resolved with a single nickel-titanium wire requires multiple aligners to correct.^54^ A more recent prospective clinical study compared apical root resorption (ARR) in fixed orthodontic appliances and clear aligners using more sensitive examinations such as cone beam computed tomography (CBCT). The authors showed that resorption was greater in patients treated with fixed orthodontic appliances than in those treated with clear aligners. The severity of resorption was significantly less in the clear aligner group than in the fixed appliance group.^55^

The prevalence of apical root resorption (ARP) in clear aligner patients remains controversial. The literature shows that the prevalence of ARR in patients treated with clear aligners is 56.30%, which is significantly lower than the fixed appliance group, which has a prevalence of 82.11%. The teeth most affected by root resorption were the maxillary canine and lateral incisor in the fixed appliance group and the least affected were the mandibular canine and lateral incisor in the clear aligner group.^54^

The presence of optimized attachments did not show a significant association with resorption, as did age and gender. A possible explanation would be that the intermittent force on the aligner allows the cementum to heal, reducing the severity of root resorption.^56^

## 9. ARE ORTHODONTIC ALIGNERS BIOCOMPATIBLE?

When we raise the possibility that materials or treatments may affect an individual’s health, we need to safely evaluate these effects. Analysis of the potential release of Bisphenol-A (BPA) from the thermoplastic material used to make Invisalign^®^ aligners showed no estrogenic and toxic effects of the aligner material on human growth factors (HGFs).^57^

A systematic review published in 2022 analyzed the release of toxic substances from 3D resins used in orthodontics and their systemic toxic effects. The review found that most toxicity studies are *in vitro* and that cytotoxic effects or estrogen levels cannot be confirmed based on limited preliminary evidence in this type of study. Although mixed results are described, 3D-printed aligners may have higher levels of cytotoxicity and genotoxicity compared to thermoplastic resins, especially those that have not undergone a final surface treatment. Therefore, clinical studies analyzing saliva, blood or even urine should be carried out to determine the levels of monomers released in humans during the use of these devices.^58^

An *in vitro* study evaluated a new light-curing resin specially produced for direct 3D printed aligners, Tera Harz TC85A (Graphy, Seoul, South Korea). During aging of these 3D-printed aligners, no factors were found that were cytotoxic to human gingival fibroblasts and did not affect their intracellular oxygen levels, and no estrogenic effects were observed.^59^

A systematic review published in 2022 analyzed 15 *in vitro* studies and found that aligner materials were considered to be slightly toxic and that cell proliferation was reduced; studies showed that aligners did not have an estrogenic effect; no monomer release could be confirmed, suggesting that the chemical is stable. The review also analyzed a randomized clinical trial that examined the levels of BPA in the saliva of 45 patients before and after using aligners, and found significant levels of BPA. Given the very high levels of BPA release observed in the single clinical study, and taking into account other possible dangers of small traces of BPA (even at low doses), as well as the numerous adverse events associated with clear aligners or clear retainers, it seems that the safety of these devices may be questionable and that more clinical biocompatibility studies are needed on this topic.^60^

A more recent systematic review, published in 2023, aimed to examine the available evidence on the release of BPA from thermoplastics used in the manufacture of clear aligners. Six studies were included in the review from a total of 1,926 records, including one randomised clinical trial and five *in vitro* studies. Only two studies found BPA release, while four reported no traces. The level of evidence ranged from low to very low. Given the conflicting evidence, the release of BPA from clear aligners cannot be confirmed or denied. Safety remains questionable until *in vivo* testing addresses this issue. In addition, it would be desirable for aligner manufacturers to be more transparent about the materials that aligners are made of, rather than keeping them a “trade secret”.^61^

## 10. WHAT IS THE EFFECT OF ORTHODONTIC ALIGNERS ON DENTAL AND PERIODONTAL STRUCTURES?

A randomized clinical trial has shown that aligners allow for better plaque control and oral hygiene^62^ because they are removable. This reduces the risk of developing white spot lesions and gingival inflammation. Therefore, orthodontic treatment with removable aligners could be recommended for patients at high risk of caries and/or periodontal disease.^63^

A systematic review found that there is a percentage of patients who develop new white spot lesions after aligner therapy, ranging from 1.2% to 41.18% in the included articles. However, the number of patients who developed white spots after aligner treatment (41.18%) is lower than those who underwent fixed orthodontic treatment with self-ligating brackets (63.64%) and conventional pre-adjusted brackets (52.94%).^64^

## 11. HOW DOES THE USE OF PLASTIC ALIGNERS AFFECT THE SUSTAINABILITY OF THE PLANET?

The environmental impact associated with plastic aligners is an aggravating factor for sustainability.^65^ ^-^ ^67^ The aligners are mainly made of PET (polyethylene terephthalate), PETG (polyethylene terephthalate glycol) or TPU (thermoplastic polyurethane), as well as other petroleum-based polymers that release a variety of nanoplastics. These components can affect marine life and ultimately cause climate change, as well as causing diseases such as immune system disorders, prostate enlargement, diabetes, hyperactivity, infertility, obesity, puberty and breast cancer in humans.^68^

Aligners can be considered an environmental hazard. As they are polymers, they cannot be disposed of as waste without special care. Their disposal must take into account the importance of these properties and the risk of contamination of the environment. There is also a risk that they may be a means of spreading cross-infection, because they have been used in the oral cavity of patients and discarded without care in the general waste. Aligners should not be subject to basic recycling, as they are classified as medical waste.^65^

A solution to this problem would be to make professionals and patients aware of the importance of collecting aligners after clinical use, using waste collectors for used aligners that can be cleaned and sent to a recycling company. This would prevent the spread of infection and ensure the correct disposal of this material as contaminated and non-biodegradable.^69^ ^,^ ^70^

Although manufacturers claim that aligners do not release BPA, recent research has found traces of this compound in both thermoplastic-based and vacuum-formed aligners. Therefore, considering the relevance of exposure to BPA, it is best to minimize or eliminate it. It would be beneficial to soak the aligner in water at 37ºC for 1 day before use, and it is extremely important to make the orthodontist aware of this matter. Consideration should be given to the proper disposal of the aligners, with a view to their safe reintroduction into the production process to protect the environment.^70^

## CONCLUSION

There is an urgent need for well-designed randomized clinical trials on orthodontic aligners. Taking into account the limitations of the currently available studies, we can conclude that:

» Considering the variety of movements performed in orthodontics, there is evidence that aligners have different levels of difficulty in performing each type of movement. Rotational and vertical movements are considered the most difficult to perform with aligners, with difficult control and low predictability. On the other hand, buccolingual and transversal movements seem to be easier to perform with these appliances. Therefore, aligners can be considered a good alternative for the orthodontic treatment of mild to moderate cases that do not involve large rotations and/or vertical movements, unless they are supplemented with auxiliary devices such as elastics, attachments and skeletal anchorage. Therefore, in complex cases, orthodontic treatment seems to be more effective when carried out with fixed appliances.
» Regarding the perception of pain caused by tooth movement, aligners seem to have less impact at the beginning of treatment. However, after the first few months of treatment, pain perception is similar between aligners and fixed appliances. On the other hand, at the beginning of treatment, aligners produce more phonoarticulatory changes than fixed appliances. When considering the other components that affect quality of life, the current evidence suggests that treatment with aligners has a similar effect to fixed appliances.
» Few studies have been conducted to assess patients’ perceptions of the aesthetic impact of orthodontic appliances. Although some studies suggest better aesthetic acceptance of aligners, orthodontists should present and discuss this issue with patients, as it is highly subjective.
» Compared to fixed appliances, aligners seem to require more treatment time in more complex cases. There is no evidence of differences in post-treatment stability between the two treatment systems.
» Evidence suggests that aligners do not prevent root resorption from occurring, but the incidence and severity of resorption may be less than that reported with fixed appliances. Another possible advantage of aligners is that they facilitate oral hygiene because they are removable, reducing the risk of white spots, tooth decay and periodontal disease.
» Tissue exposure to the release of isocyanate from plastic aligners can cause adverse oral health effects, such as allergic contact reactions, and we still have dysphagia and salivary flow, which increase when the patient uses removable appliances; and this may be considered unfavourable. Given the conflicting evidence, the release of BPA from clear aligners cannot be denied. Safety remains questionable until high-quality *in vivo* testing provides better evidence on this issue.
» Because they are made of plastic, and because of the risks associated with this material, manufacturers and orthodontists need to find solutions that reduce the environmental impact of discarding aligners.

## References

[1] Lagravère MO, Flores-Mir C (2005). The treatment effects of Invisalign orthodontic aligners a systematic review. J Am Dent Assoc.

[2] Papadimitriou A, Mousoulea S, Gkantidis N, Kloukos D (2018). Clinical effectiveness of Invisalign(r) orthodontic treatment a systematic review. Prog Orthod.

[3] Galan-Lopez L, Barcia-Gonzalez J, Plasencia E (2019). A systematic review of the accuracy and efficiency of dental movements with Invisalign(r) Korean. J Orthod.

[4] Robertson L, Kaur H, Fagundes NCF, Romanyk D, Major P, Flores-Mir C (2020). Effectiveness of clear aligner therapy for orthodontic treatment A systematic review. Orthod Craniofac Res.

[5] Jaber ST, Hajeer MY, Burhan AS (2022). The effectiveness of in-house clear aligners and traditional fixed appliances in achieving good occlusion in complex orthodontic cases a randomized control clinical trial. Cureus.

[6] Rossini G, Parrini S, Castroflorio T, Deregibus A, Debernardi CL (2015). Efficacy of clear aligners in controlling orthodontic tooth movement a systematic review. Angle Orthod.

[7] Nucera R, Dolci C, Bellocchio AM, Costa S, Barbera S, Rustico L (2022). Effects of composite attachments on orthodontic clear aligners therapy a systematic review. Materials.

[8] Invisalign (2023). O Invisalign é indicado para mim.

[9] Upadhyay M, Arqub SA (2022). Biomechanics of clear aligners hidden truths & first principles. J World Fed Orthod.

[10] Yassir YA, Nabbat SA, McIntyre GT, Bearn DR (2022). Clinical effectiveness of clear aligner treatment compared to fixed appliance treatment an overview of systematic reviews. Clin Oral Investig.

[11] Goh S, Dreyer C, Weir T (2023). The predictability of the mandibular curve of Wilson, buccolingual crown inclination, and transverse expansion expression with Invisalign treatment. Am J Orthod Dentofacial Orthop.

[12] Cretella Lombardo E, Paoloni V, Fanelli S, Pavoni C, Gazzani F, Cozza P (2022). Evaluation of the upper arch morphological changes after two different protocols of expansion in early mixed dentition rapid maxillary expansion and Invisalign(r) First system. Life.

[13] Tien R, Patel V, Chen T, Lavrin I, Naoum S, Lee RJH (2023). The predictability of expansion with Invisalign a retrospective cohort study. Am J Orthod Dentofacial Orthop.

[14] Perrotti G, Carrafiello A, Rossi O, Karanxha L, Baccaglione G, Del Fabbro M (2022). Clinical use of aligners associated with Nuvola(r) OP system for transverse maxillary deficiency a retrospective study on 100 patients. Int J Environ Res Public Health.

[15] Levrini L, Carganico A, Abbate L (2021). Maxillary expansion with clear aligners in the mixed dentition a preliminary study with Invisalign(r) First system. Eur J Paediatr Dent.

[16] Lione R, Paoloni V, De Razza FC, Pavoni C, Cozza P (2022). The efficacy and predictability of maxillary first molar derotation with Invisalign a prospective clinical study in growing subjects. Appl Sci.

[17] Bilello G, Fazio M, Amato E, Crivello L, Galvano A, Currò G (2022). Accuracy evaluation of orthodontic movements with aligners a prospective observational study. Prog Orthod.

[18] Lione R, Paoloni V, De Razza FC, Pavoni C, Cozza P (2022). Analysis of maxillary first molar derotation with Invisalign clear aligners in permanent dentition. Life.

[19] Castroflorio T, Sedran A, Parrini S, Garino F, Reverdito M, Capuozzo R (2023). Predictability of orthodontic tooth movement with aligners effect of treatment design. Prog Orthod.

[20] Blundell HL, Weir T, Byrne G (2022). Predictability of overbite control with the Invisalign appliance comparing SmartTrack with precision bite ramps to EX30. Am J Orthod Dentofacial Orthop.

[21] Suh H, Garnett BS, Mahood K, Mahjoub N, Boyd RL, Oh H (2022). Treatment of anterior open bites using non-extraction clear aligner therapy in adult patients. Korean J Orthod.

[22] Harris K, Ojima K, Dan C, Upadhyay M, Alshehri A, Kuo CL (2020). Evaluation of open bite closure using clear aligners a retrospective study. Prog Orthod.

[23] Hennessy J, Garvey T, Al-Awadhi EA (2016). A randomized clinical trial comparing mandibular incisor proclination produced by fixed labial appliances and clear aligners. Angle Orthod.

[24] Rossini G, Parrini S, Castroflorio T, Deregibus A, Debernardi CL (2015). Efficacy of clear aligners in controlling orthodontic tooth movement a systematic review. Angle Orthod.

[25] Barreda GJ, Dzierewianko EA, Muñoz KA, Piccoli GI (2017). Surface wear of resin composites used for Invisalign(r) attachments. Acta Odontol Latinoam.

[26] Putrino A, Barbato E, Galluccio G (2021). Clear aligners between evolution and efficiency: a scoping review. Int J Environ Res Public Health.

[27] Savignano R, Valentino R, Razionale AV, Michelotti A, Barone S, D'Antò V (2019). Biomechanical effects of different auxiliary-aligner designs for the extrusion of an upper central incisor a finite element analysis. J Healthc Eng.

[28] Dasy H, Dasy A, Asatrian G, Rózsa N, Lee HF, Kwak JH (2015). Effects of variable attachment shapes and aligner material on aligner retention. Angle Orthod.

[29] Rongo R Dianišková S, Spiezia A Bucci R, Michelotti A D’Antò V (2022). Class II malocclusion in adult patients what are the effects of the intermaxillary elastics with clear aligners? A retrospective single center one-group longitudinal study. J Clin Med.

[30] Roberts WE, Chang CH, Chen J, Brezniak N, Yadav S (2022). Integrating skeletal anchorage into fixed and aligner biomechanics. J World Fed Orthod.

[31] Papageorgiou SN, Koletsi D, Iliadi A, Peltomaki T, Eliades T (2020). Treatment outcome with orthodontic aligners and fixed appliances a systematic review with meta-analyses. Eur J Orthod.

[32] Gaffuri F, Cossellu G, Lanteri V, Brotto E, Farronato M (2020). Comparative effectiveness of Invisalign and fixed appliances in first-premolar extraction cases. J Clin Orthod.

[33] Graf I, Puppe C, Schwarze J, Höfer K, Christ H, Braumann B (2021). Evaluation of effectiveness and stability of aligner treatments using the Peer Assessment Rating Index. J Orofac Orthop.

[34] Silva VM, Ayub PV, Massaro C, Janson G, Garib D (2023). Comparison between clear aligners and 2 × 4 mechanics in the mixed dentition a randomized clinical trial. Angle Orthod.

[35] Jawaid M, Qadeer TA (2019). Assessment of the changes in the oral health related quality of life 24 hours following insertion of fixed orthodontic appliance components - An observational cross-sectional study conducted at Bahria University Medical and Dental College Karachi. J Pak Med Assoc.

[36] Costa AA, Ferreira MC, Serra-Negra JM, Pordeus IA, Paiva SM (2011). Impact of wearing fixed orthodontic appliances on oral health-related quality of life among Brazilian children. J Orthod.

[37] Zhang B, Huang X, Huo S, Zhang C, Zhao S, Cen X (2020). Effect of clear aligners on oral health-related quality of life a systematic review. Orthod Craniofac Res.

[38] AlSeraidi M, Hansa I, Dhaval F, Ferguson DJ, Vaid NR (2021). The effect of vestibular, lingual, and aligner appliances on the quality of life of adult patients during the initial stages of orthodontic treatment. Prog Orthod.

[39] Cardoso PC, Espinosa DG, Mecenas P, Flores-Mir C, Normando D (2020). Pain level between clear aligners and fixed appliances a systematic review. Prog Orthod.

[40] Alcón S, Curto A, Alvarado M, Albaladejo A, Garcovich D, Alvarado-Lorenzo A (2021). Comparative analysis of periodontal pain using two different orthodontic techniques, fixed multibrackets and removable aligners a longitudinal clinical study with monthly follow-ups for 12 months. Appl Sci.

[41] Rai AK, Rozario JE, Ganeshkar SV (2014). Comparison of speech performance in labial and lingual orthodontic patients a prospective study. Dent Res J.

[42] Ali Baeshen H, El-Bialy T, Alshehri A, Awadh W, Thomas J, Dhillon H (2023). The effect of clear aligners on speech a systematic review. Eur J Orthod.

[43] Khattab TZ, Farah H, Al-Sabbagh R, Hajeer MY, Haj-Hamed Y (2013). Speech performance and oral impairments with lingual and labial orthodontic appliances in the first stage of fixed treatment. Angle Orthod.

[44] Hohoff A, Seifert E, Fillion D, Stamm T, Heinecke A, Ehmer U (2003). Speech performance in lingual orthodontic patients measured by sonagraphy and auditive analysis. Am J Orthod Dentofacial Orthop.

[45] Pogal-Sussman-Gandia CB, Tabbaa S, Al-Jewair T (2019). Effects of Invisalign(r) treatment on speech articulation. Int Orthod.

[46] Wang D, Firth F, Bennani F, Farella M, Mei L (2023). Immediate effect of clear aligners and fixed appliances on perioral soft tissues and speech. Orthod Craniofac Res.

[47] Fraundorf EC, Araújo E, Ueno H, Schneider PP, Kim KB (2022). Speech performance in adult patients undergoing Invisalign treatment. Angle Orthod.

[48] Thai JK, Araujo E, McCray J, Schneider PP, Kim KB (2020). Esthetic perception of clear aligner therapy attachments using eye-tracking technology. Am J Orthod Dentofacial Orthop.

[49] Pacheco-Pereira C, Brandelli J, Flores-Mir C (2018). Patient satisfaction and quality of life changes after Invisalign treatment. Am J Orthod Dentofacial Orthop.

[50] Alami S, Sahim S, Hilal F, Essamlali A, El Quars F (2022). Perception and satisfaction of patients treated with orthodontic clear aligners. Open Access Lib J.

[51] Zheng M, Liu R, Ni Z, Yu Z (2017). Efficiency, effectiveness and treatment stability of clear aligners a systematic review and meta-analysis. Orthod Craniofac Res.

[52] Elhaddaoui R, Qoraich HS, Bahije L, Zaoui F (2017). Orthodontic aligners and root resorption A systematic review. Int Orthod.

[53] Fang X, Qi R, Liu C (2019). Root resorption in orthodontic treatment with clear aligners a systematic review and meta-analysis. Orthod Craniofac Res.

[54] Jyotirmay, Singh SK, Adarsh K, Kumar A, Gupta AR, Sinha A (2021). Comparison of apical root resorption in patients treated with fixed orthodontic appliance and clear aligners a cone-beam computed tomography study. J Contemp Dent Pract.

[55] Li Y, Deng S, Mei L, Li Z, Zhang X, Yang C (2020). Prevalence and severity of apical root resorption during orthodontic treatment with clear aligners and fixed appliances a cone beam computed tomography study. Prog Orthod.

[56] Liu W, Shao J, Li S, Al-Balaa M, Xia L, Li H (2021). Volumetric cone-beam computed tomography evaluation and risk factor analysis of external apical root resorption with clear aligner therapy. Angle Orthod.

[57] Ahrari F, Tavakkol Afshari J, Poosti M, Brook A (2010). Cytotoxicity of orthodontic bonding adhesive resins on human oral fibroblasts. Eur J Orthod.

[58] Francisco I, Paula AB, Ribeiro M, Marques F, Travassos R, Nunes C (2022). The biological effects of 3D resins used in orthodontics a systematic review. Bioengineering.

[59] Pratsinis H, Papageorgiou SN, Panayi N, Iliadi A, Eliades T, Kletsas D (2022). Cytotoxicity and estrogenicity of a novel 3-dimensional printed orthodontic aligner. Am J Orthod Dentofacial Orthop.

[60] Yazdi M, Daryanavard H, Ashtiani AH, Moradinejad M, Rakhshan V (2023). A systematic review of biocompatibility and safety of orthodontic clear aligners and transparent vacuum-formed thermoplastic retainers bisphenol-A release, adverse effects, cytotoxicity, and estrogenic effects. Dent Res J.

[61] Peter E, Monisha J, George SA (2023). Bisphenol-A release from thermoplastic clear aligner materials A systematic review. J Orthod.

[62] Kannan A, Padmanabhan S (2019). Comparative evaluation of Icon(r) resin infiltration and Clinpro(tm) XT varnish on colour and fluorescence changes of white spot lesions a randomized controlled trial. Prog Orthod.

[63] Albhaisi Z, Al-Khateeb SN, Abu Alhaija ES (2020). Enamel demineralization during clear aligner orthodontic treatment compared with fixed appliance therapy, evaluated with quantitative light-induced fluorescence a randomized clinical trial. Am J Orthod Dentofacial Orthop.

[64] Abay F, Buyuk SK, Korkmaz YN (2022). Prevalence of white spot lesions during clear aligner therapy a systematic review. Aust Orthod J.

[65] Freitas MPM (2022). Aligners, environmental contamination, and the role of orthodontics. Angle Orthod.

[66] Bichu YM, Alwafi A, Liu X, Andrews J, Ludwig B, Bichu AY (2022). Advances in orthodontic clear aligner materials. Bioact Mater.

[67] Peter E, Monisha J, Ani George S (2022). Are clear aligners environment friendly. Am J Orthod Dentofacial Orthop.

[68] Gibson RL (2007). Toxic baby bottles: scientific study finds leaching chemicals in clear plastic baby bottles.

[60] Iliadi A, Koletsi D, Papageorgiou SN, Eliades T (2020). Safety considerations for thermoplastic-type appliances used as orthodontic aligners or retainers a systematic review and meta-analysis of clinical and in-vitro research. Materials.

[70] Iliadi A, Koletsi D, Papageorgiou SN, Eliades T (2020). Safety considerations for thermoplastic-type appliances used as orthodontic aligners or retainers a systematic review and meta-analysis of clinical and in-vitro research. Materials (Basel).

